# Supplementary material for: Relationship between the Molecular Geometry and the Radiative Efficiency in Naphthyl-Based Bis-Ortho-Carboranyl Luminophores
Source: Molecules. 2022 Oct 4;27(19):6565. doi: 10.3390/molecules27196565 (PMC9572229; doi:10.3390/molecules27196565)
Supplement: Supplementary file 1 [file molecules-27-06565-s001.zip › molecules-1927776-supplementary.pdf]

Supplementary Material

Relationship Between the Molecular Geometry and the Radiative Efficiency in Naphthyl-based Bis-*ortho*-Carboranyl Luminophores

Sanghee Yi,[1,†] Mingi Kim[1,†] Ju Hyun Hong,[1] Yung Ju Seo,[1] and Kang Mun Lee[1,*]

[1] Department of Chemistry, Institute for Molecular Science and Fusion Technology, Kangwon National University, Chuncheon 24341, Republic of Korea.

*Correspondence: kangmunlee@kangwon.ac.kr (K.M.L.); Tel.: +82-33-250-8499 (K.M.L)
[†]The first and second authors contributed equally to this work.

Contents

Multinuclear NMR Spectra for *o*-carboranyl compounds and their precursors ·············· S2–S11

Crystallographic data and parameters for *o*-carboranyl compounds ··························· S12

Selected bond lengths (Å) and angles (°) for *o*-carboranyl compounds ······················· S13

UV-vis absorption and PL spectra for naphthalene ··· S14

Emission decay curves for **15CS** and **26CS** in film state ·· S15

Theoretical calculation details for *o*-carboranyl compounds ···································· S16–S31

Cartesian coordinates of *o*-carboranyl compounds in each optimized geometry ············· S32–S39

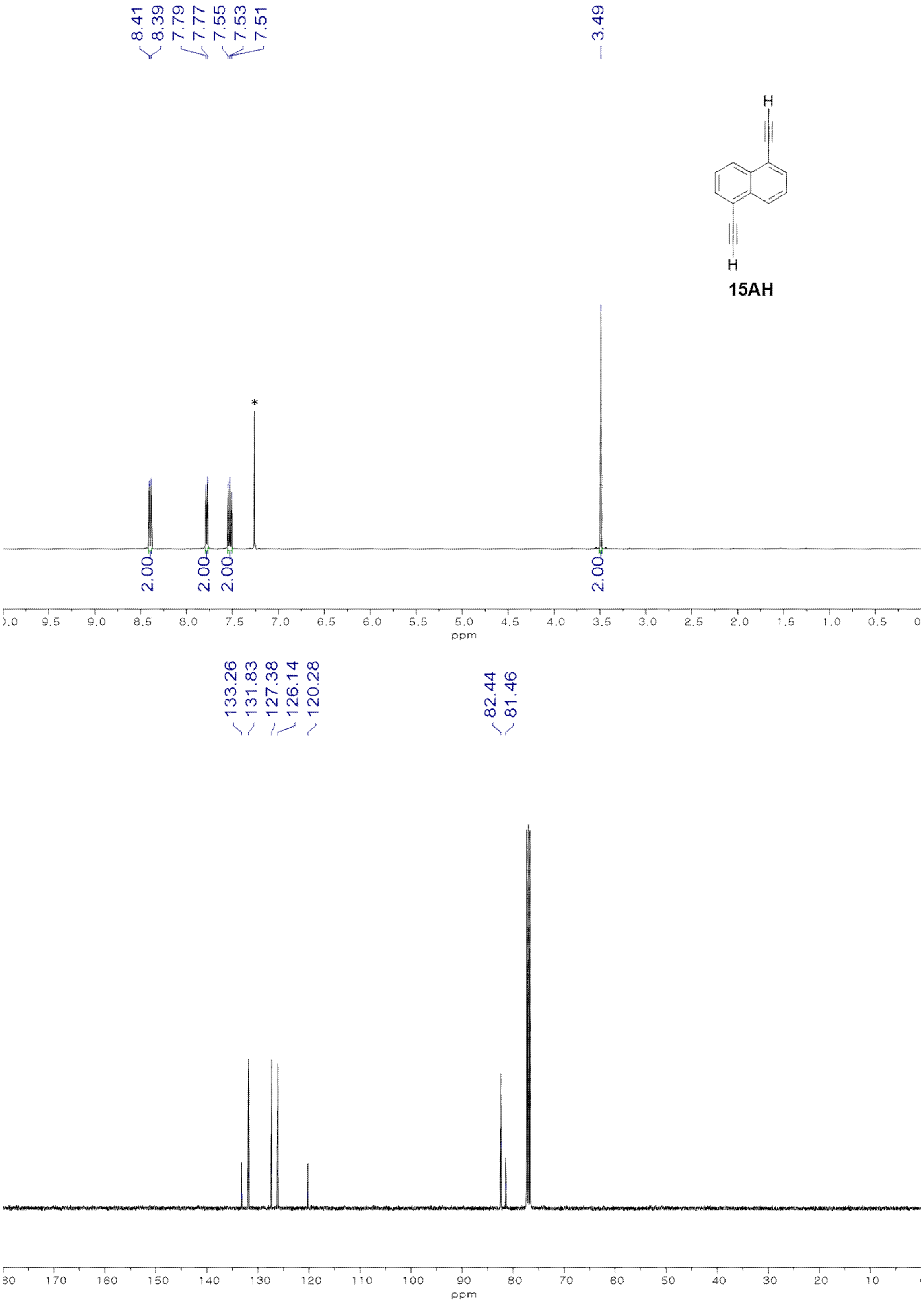

Figure S1. ^{1}H (top) and ^{13}C (bottom) NMR spectra of **15AH** in $CDCl_3$ (* from residual $CHCl_3$ in $CDCl_3$).

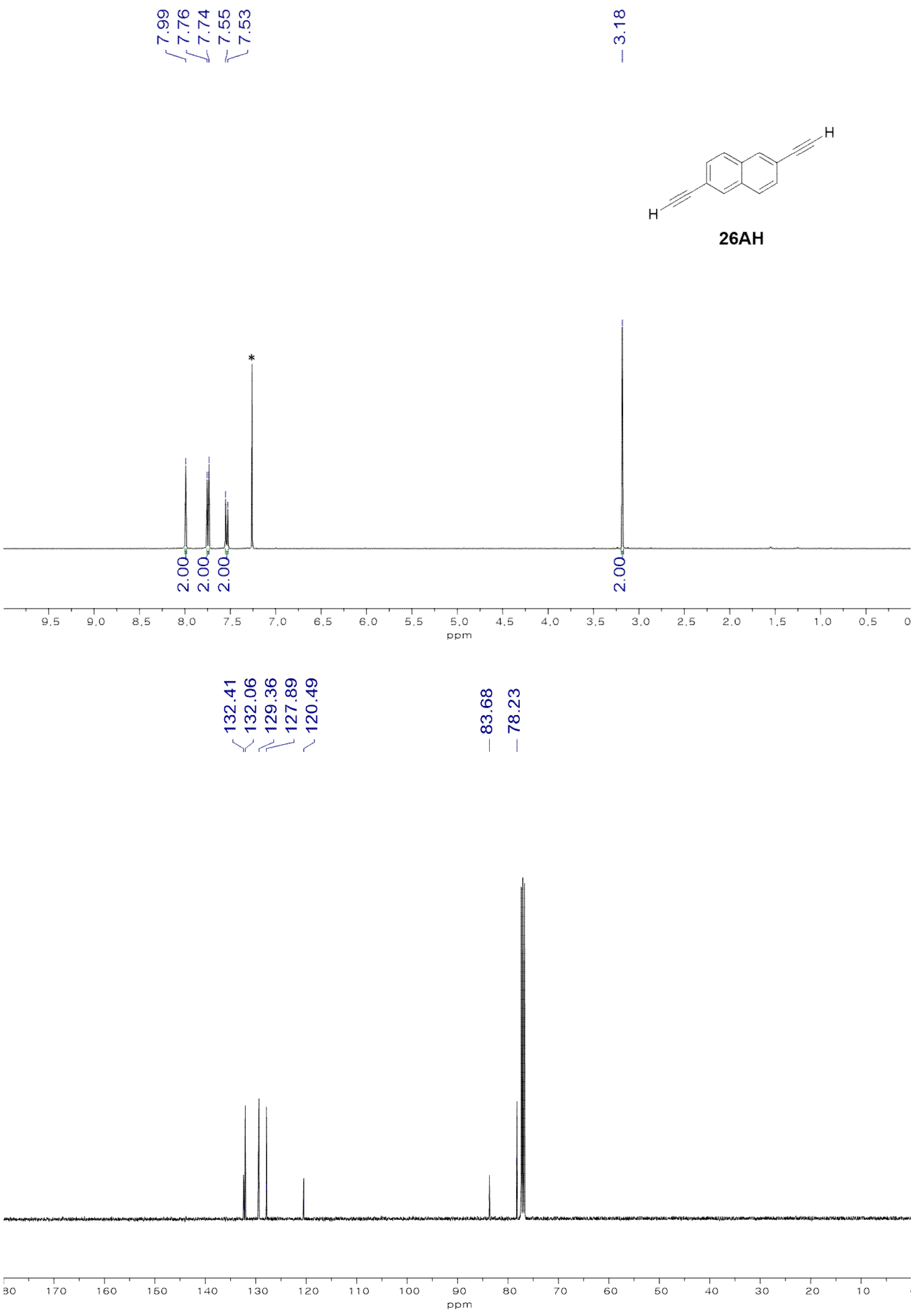

Figure S2. ^{1}H (top) and ^{13}C (bottom) NMR spectra of **26AH** in $CDCl_3$ (* from residual $CHCl_3$ in $CDCl_3$).

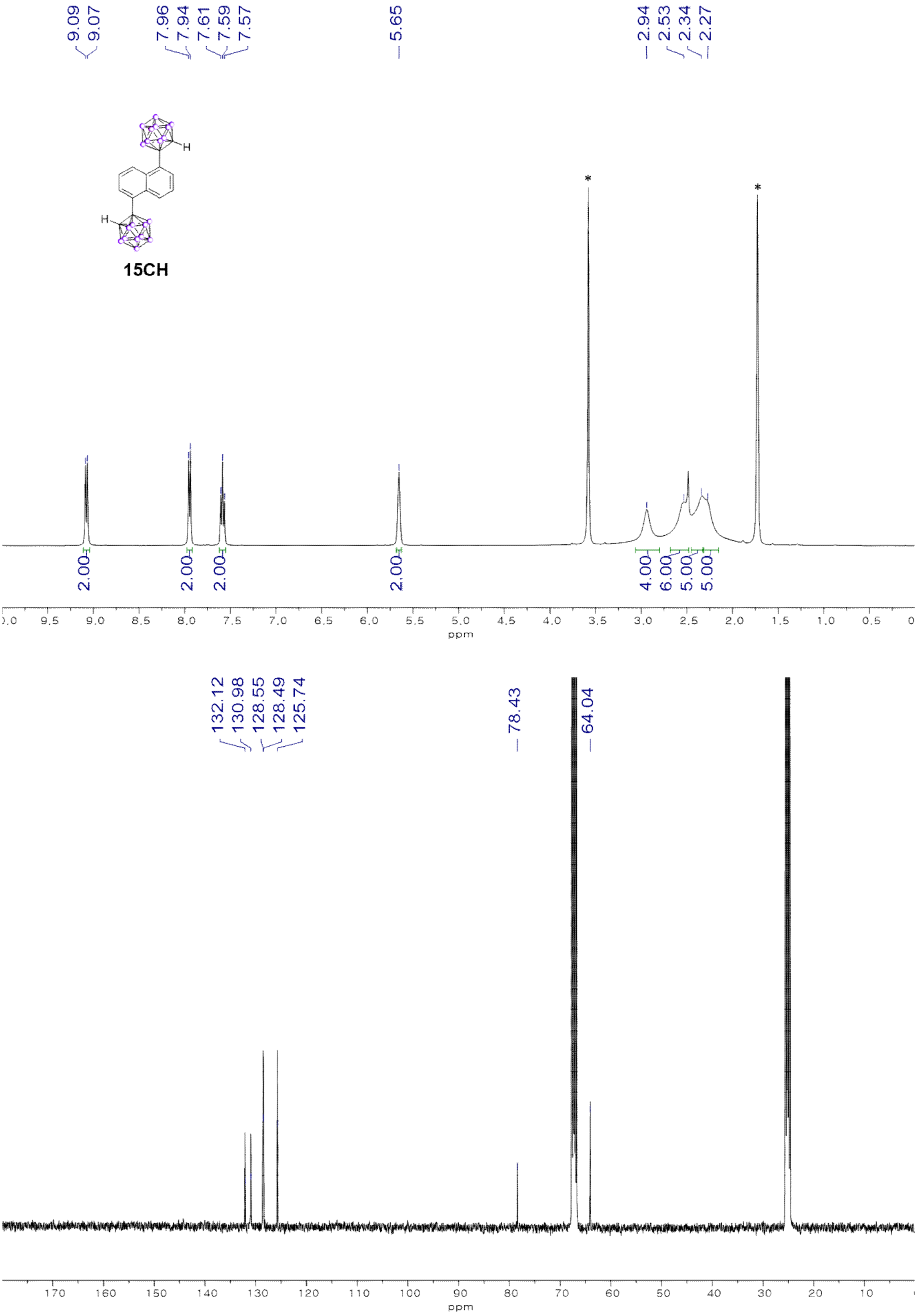

Figure S3. $^{1}H\{^{11}B\}$ (top) and ^{13}C (bottom) NMR spectra of **15CH** in THF-d^8 (* from residual THF in THF-d^8).

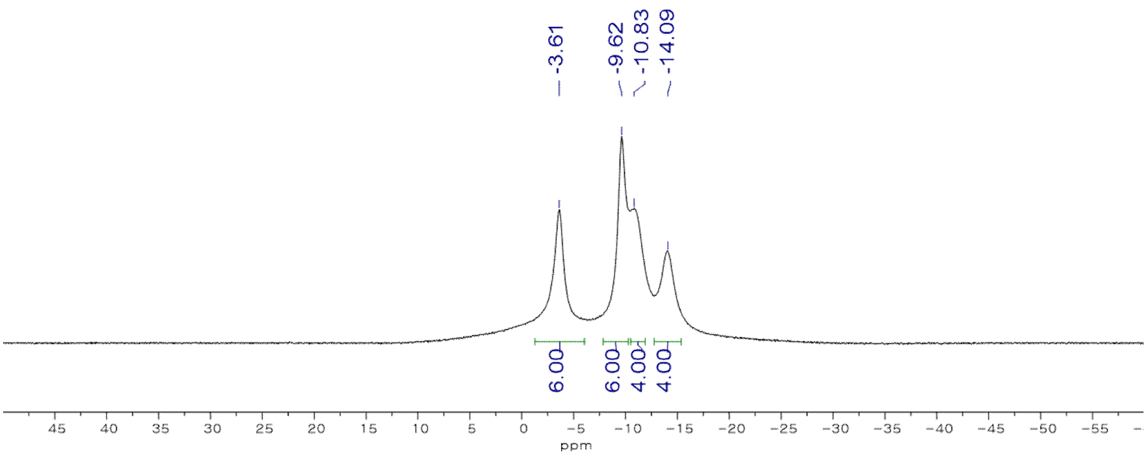

Figure S4. $^{11}B\{^{1}H\}$ NMR spectra of **15CH** in THF-d^8.

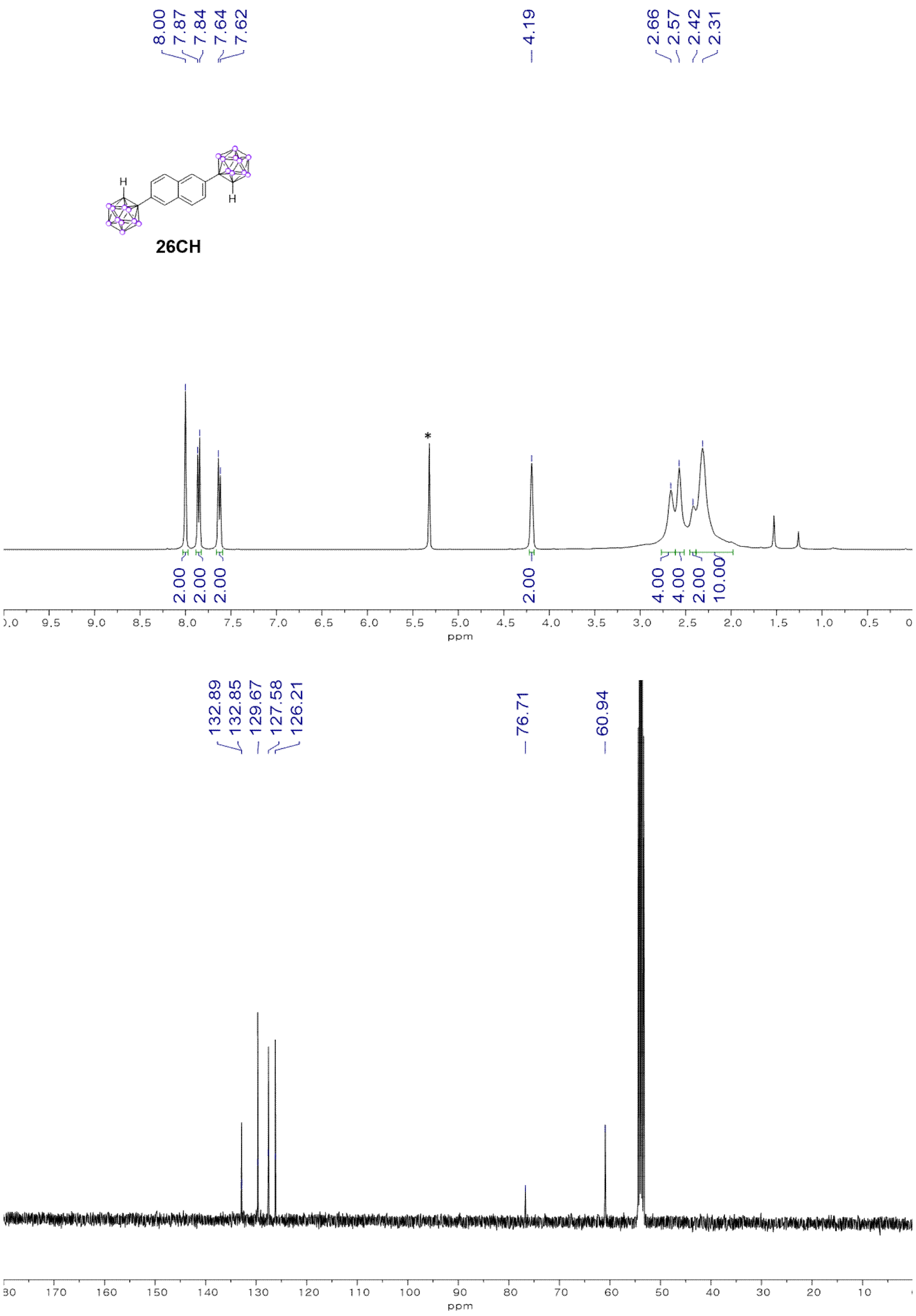

Figure S5. $^1H\{^{11}B\}$ (top) and ^{13}C (bottom) NMR spectra of **26CH** in CD_2Cl_2 (* from residual CH_2Cl_2 in CD_2Cl_2).

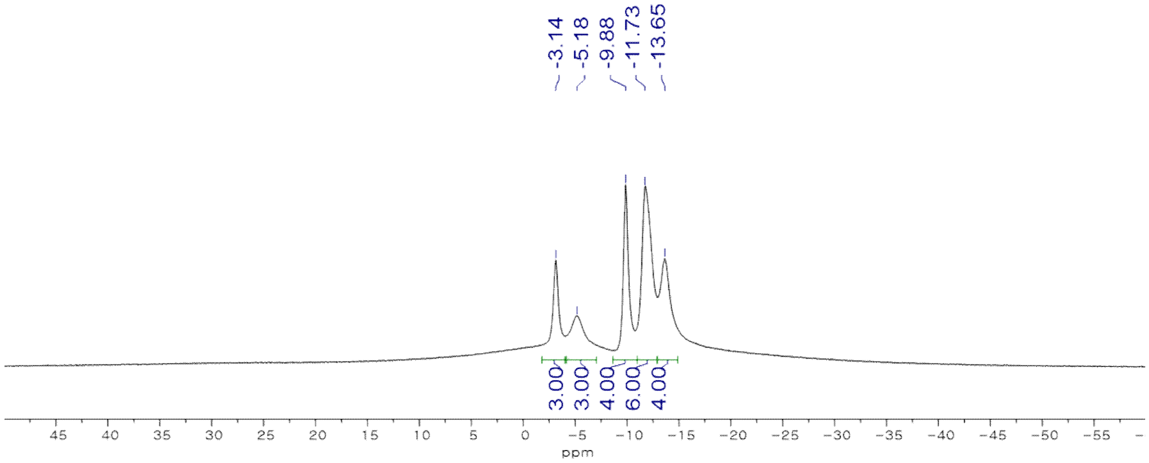

Figure S6. $^{11}B\{^1H\}$ NMR spectra of **26CH** in CD_2Cl_2.

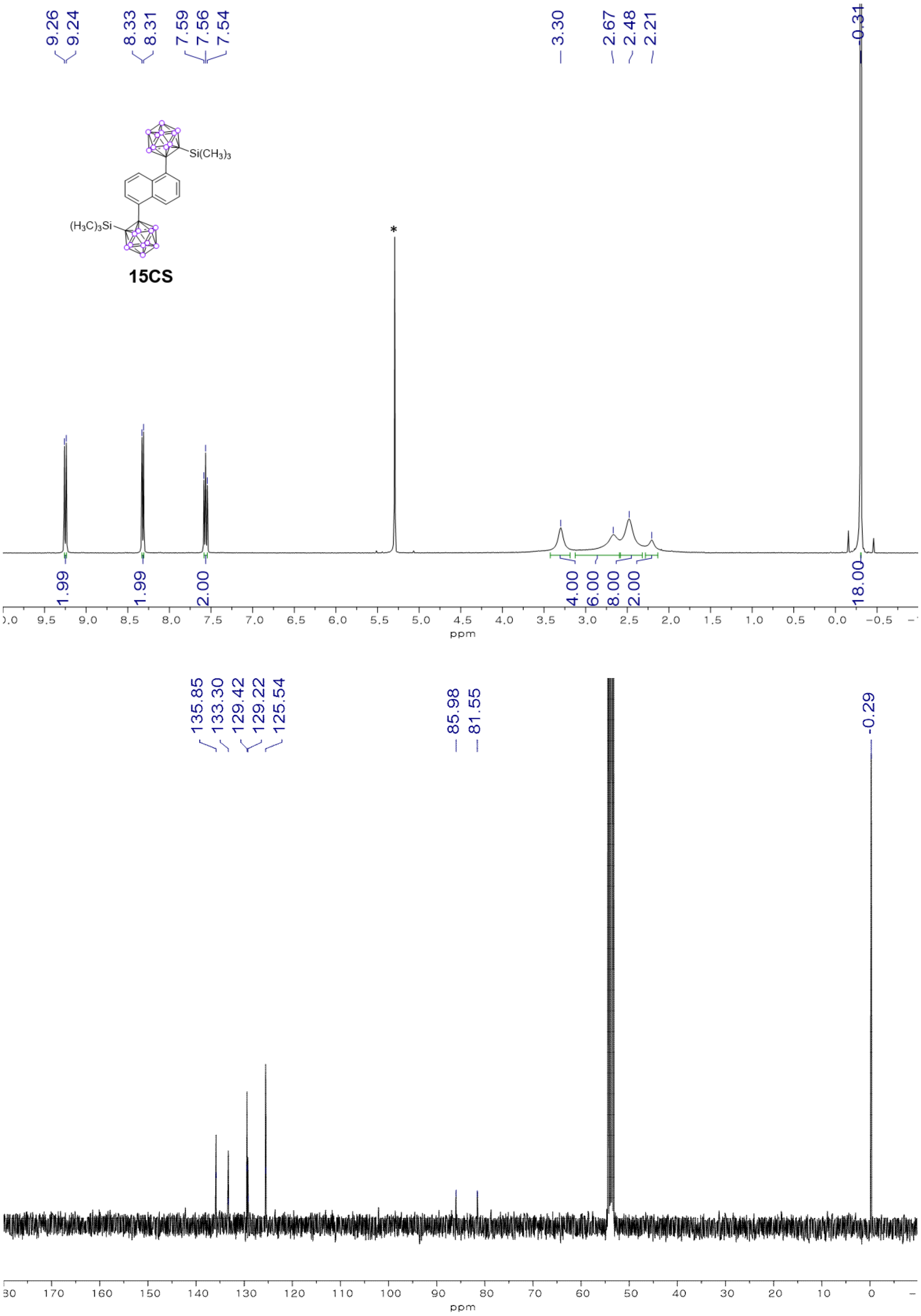

Figure S7. $^{1}H\{^{11}B\}$ (top) and ^{13}C (bottom) NMR spectra of **15CS** in CD_2Cl_2 (* from residual CH_2Cl_2 in CD_2Cl_2).

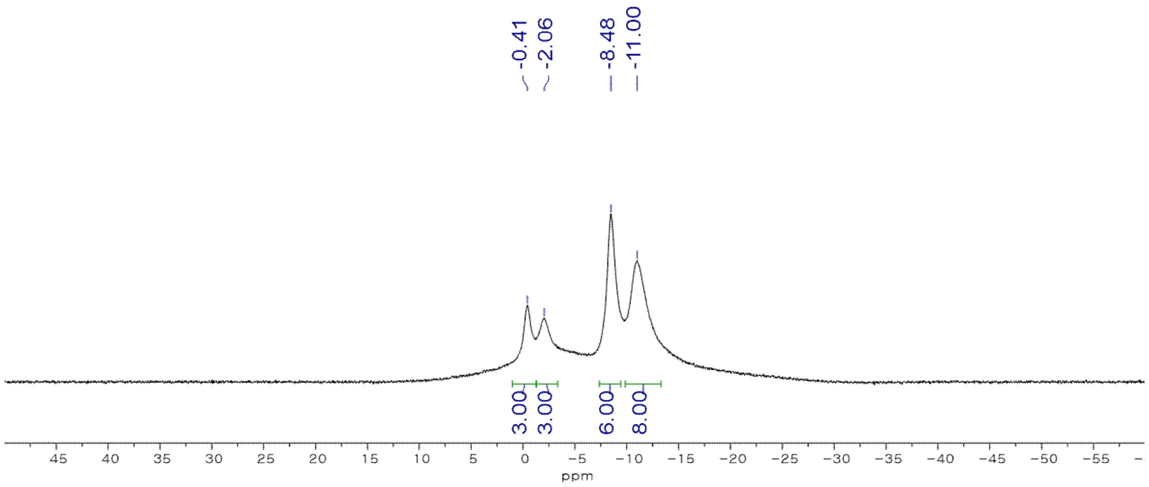

Figure S8. $^{11}B\{^1H\}$ NMR spectra of **15CS** in CD_2Cl_2.

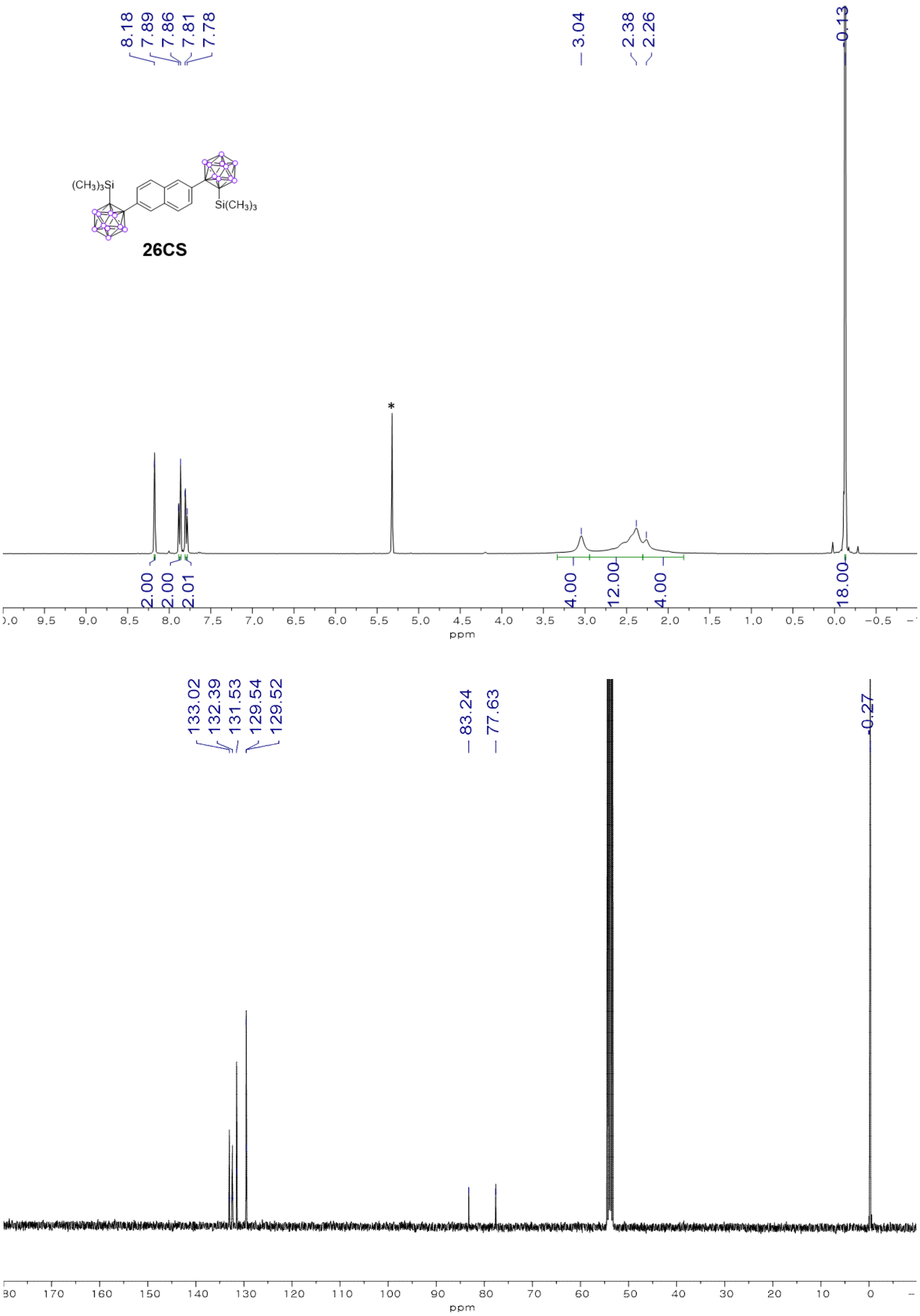

Figure S9. $^{1}H\{^{11}B\}$ (top) and ^{13}C (bottom) NMR spectra of **26CS** in CD_2Cl_2 (* from residual CH_2Cl_2 in CD_2Cl_2).

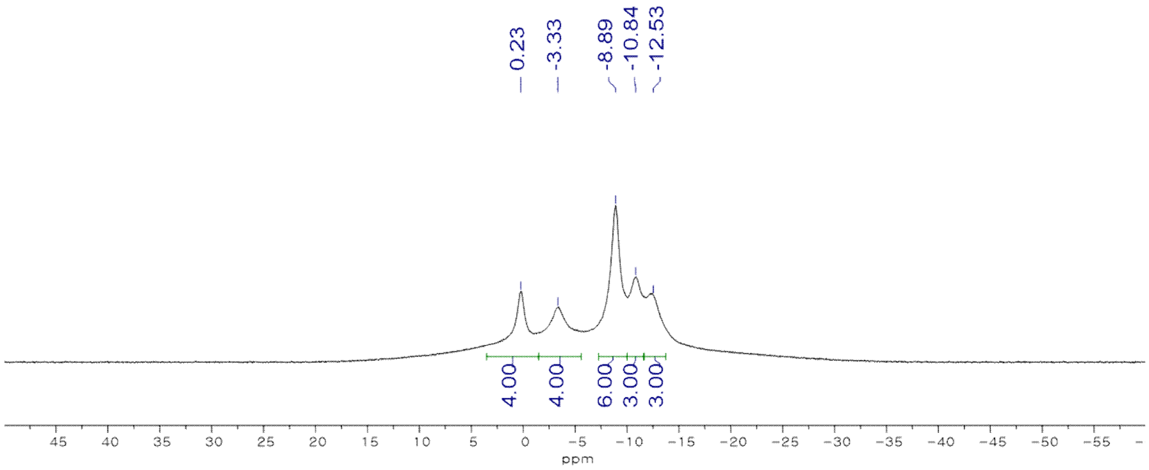

Figure S10. $^{11}B\{^{1}H\}$ NMR spectra of **26CS** in CD_2Cl_2.

Table S1. Crystallographic parameters for **15CH**, **26CH**, **15CS**, and **26CS**

Compound	**15CH**	**26CH**	**15CS**	**26CS**
Formula	$C_{14}H_{28}B_{20}$	$C_{14}H_{28}B_{20}$	$C_{20}H_{44}B_{20}Si_2$	$C_{20}H_{44}B_{20}Si_2$
Fomula weight	412.56	412.56	556.93	556.93
Crystal system	Monoclinic	Monoclinic	Triclinic	Triclinic
Space group	$P2_1/n$	$P2_1/n$	P_{-1}	P_{-1}
a (Å)	7.760(2)	7.0213(5)	6.8599(3)	6.8075(7)
b (Å)	11.251(3)	21.8080(15)	9.1226(4)	9.0053(11)
c (Å)	13.293(4)	7.8763(5)	13.9059(6)	13.2536(16)
α (°)	90	90	98.6628(17)	89.541(5)
β (°)	90.923(9)	90.163(2)	95.9771(17)	89.729(5)
γ (°)	90	90	105.4768(17)	83.481(5)
V (Å^3)	1160.5(6)	1206.02(14)	819.54(6)	807.21(16)
Z	2	2	1	1
ρ_{calc}(g cm^{-3})	1.181	1.136	1.128	1.146
μ (mm^{-1})	0.054	0.052	0.123	0.125
F(000)	424	424	292	292
T (K)	296(2)	296(2)	296(2)	173(2)
Scan mode	*φ and ω-scan*	*φ and ω-scan*	*φ and ω-scan*	*φ and ω-scan*
hkl range	–8 → +9,<br>–12 → +13,<br>–15 → +15	–8 → +9,<br>–28 → +29,<br>–10 → +8	–9 → +9,<br>–12 → +12,<br>–18 → +18	–8 → +8<br>–11 → +11,<br>–17 → +17
Measd reflns	14219	14536	20655	14128
Unique reflns [R_{int}]	2108 [0.1107]	2872 [0.0552]	4038 [0.0410]	3571 [0.0742]
Reflns used for refinement	2108	2872	4038	3571
Refined parameters	160	308	196	194
R_1[1] (I > 2σ(I))	0.1251	0.0674	0.0429	0.0843
wR_2[2] all data	0.2213	0.1979	0.1292	0.2296
GOF on F^2	1.252	1.026	1.005	1.025
ρ_{fin} (max/min) (e Å^{-3})	0.261, –0.218	0.141, –0.177	0.273, –0.217	0.591, –0.576

[1]$R_1 = \sum||Fo| - |Fc||/\sum|Fo|$. [2]$wR_2 = \{[\sum w(Fo^2 - Fc^2)^2]/[\sum w(Fo^2)^2]\}^{1/2}$.

Table S2. Selected bond lengths (Å) and angles (°) for **15CH**, **26CH**, **15CS**, and **26CS**

	**15CH**	**26CH**	**15CS**	**26CS**
bond lengths /Å				
C1–C15	1.529(6)	–	1.5259(16)	–
C2–C15	–	1.469(15)	–	1.515(5)
C15–C16	1.674(6)	1.567(15)	1.7411(16)	1.704(4)
C16–Si1	–	–	1.9326(13)	1.926(4)
angles /°				
C1–C15–C16	117.0(3)	–	119.77(9)	–
C2–C15–C16	–	117.8(8)	–	119.8(3)
C15–C16–H101	116(3)	118.1	–	–
C15–C16–Si1	–	–	121.85(8)	122.2(2)

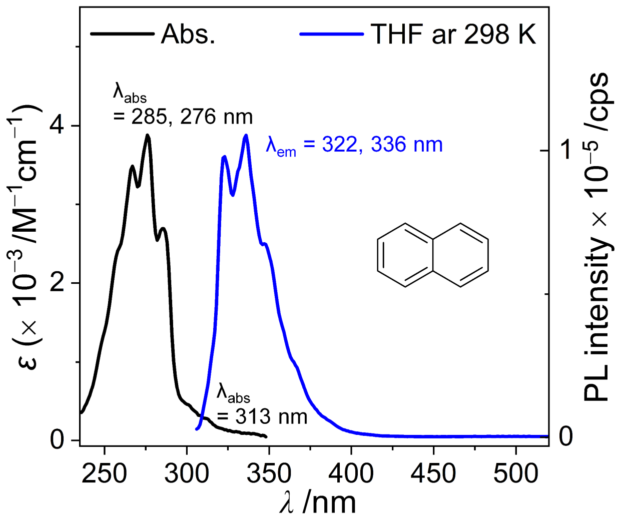

Figure S11. UV-vis absorption (left side) and PL spectra (right side) for naphthalene in THF (50 μM, λ_{ex} = 267 nm).

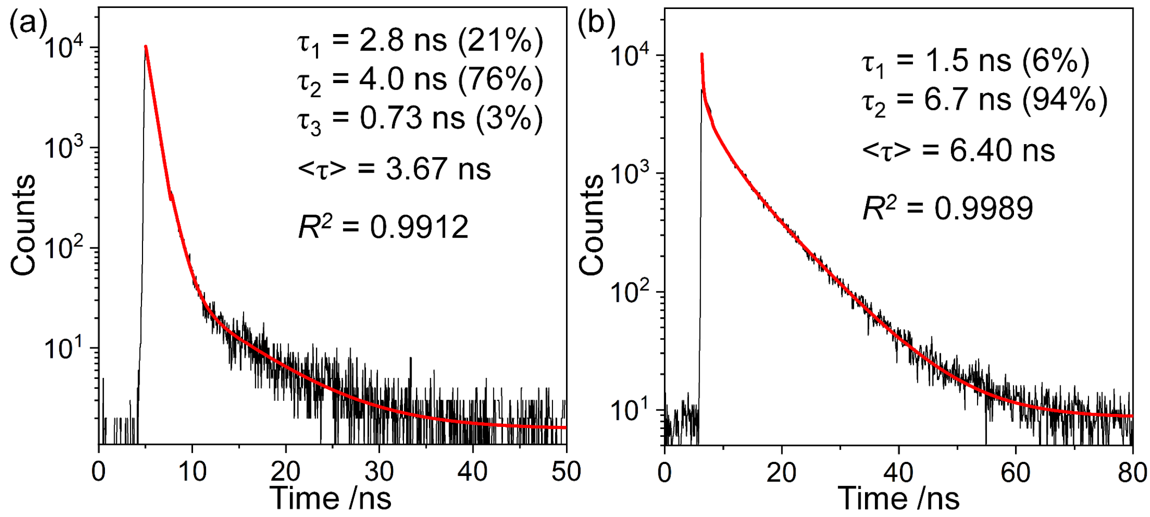

Figure S12. Emission decay curves for (a) **15CS** and (b) **26CS** in film detected at each emissive maxima at 298 K. Each red-line is its exponential fitting curve for the decay curves.

Theoretical calculation details

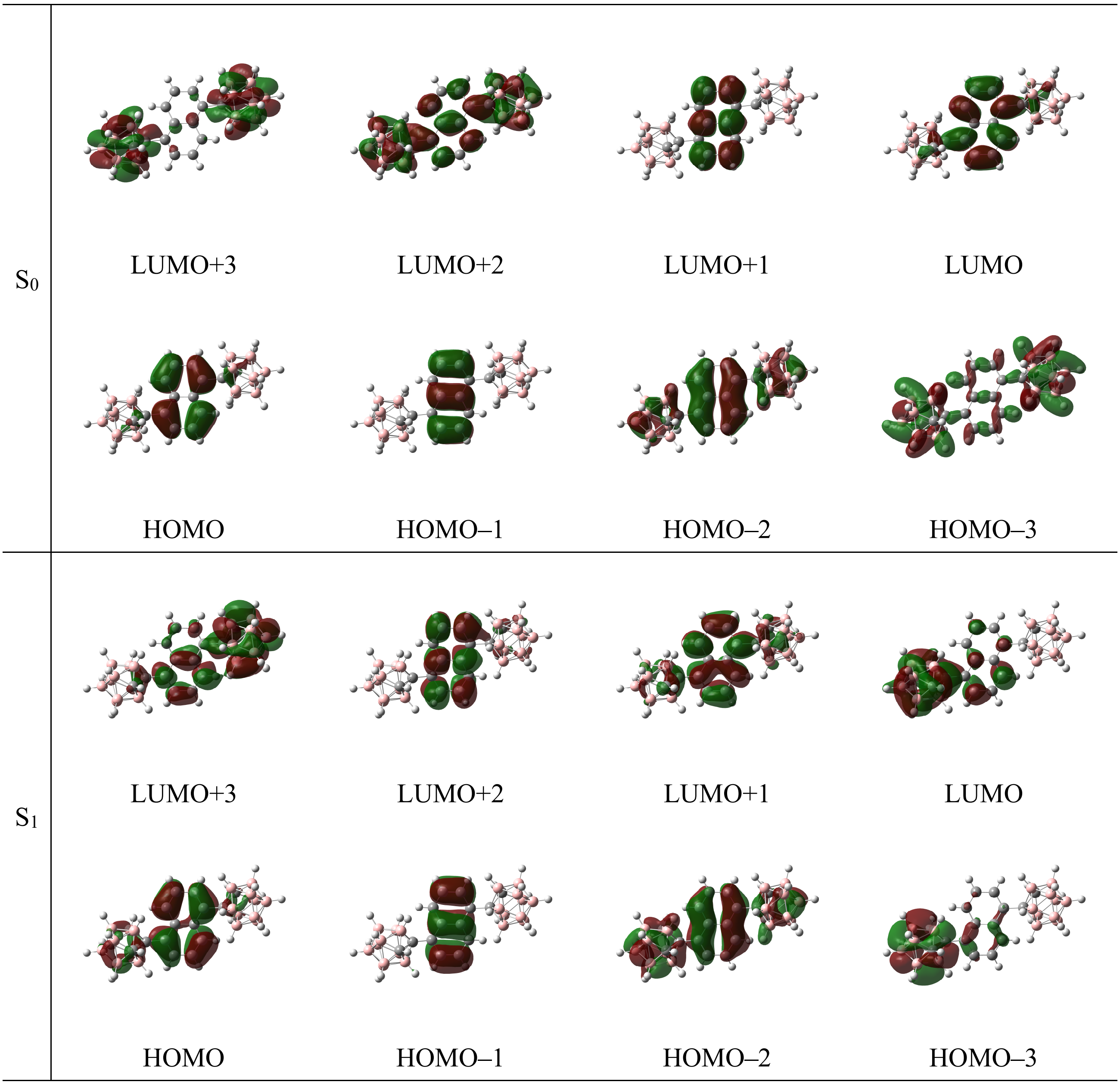

Figure S13. The selected frontier orbitals of **15CH** from TD-B3LYP calculations (Isovalue = 0.04 a.u.) at the ground state (S_0) and first singlet excited state (S_1) optimized geometries in THF.

Table S3. Computed absorption wavelengths (λ_{calc} in nm) and oscillator strengths ($f_{calc.}$) for **15CH** from TD-B3LYP calculations using the B3LYP geometries at the ground state (S_0) and first singlet excited state (S_1) optimized geometries in THF

state	λ_{calc} /nm	f_{calc}	Major contribution		
			S_0		
1	307.83	0.4111	HOMO	→	LUMO (96.03%)
2	286.12	0.0135	HOMO–1	→	LUMO (61.34%)
			HOMO	→	LUMO+1 (36.99%)
3	247.88	0.0000	HOMO–2	→	LUMO (11.88%)
			HOMO	→	LUMO+2 (87.00%)
4	228.91	0.7796	HOMO–1	→	LUMO (33.77%)
			HOMO	→	LUMO+1 (56.93%)
5	227.86	0.0000	HOMO–2	→	LUMO (26.65%)
			HOMO	→	LUMO+3 (67.22%)
			S_1		
1	545.00	0.4473	HOMO	→	LUMO (99.68%)
2	398.29	0.0227	HOMO–1	→	LUMO (97.38%)
3	371.39	0.0009	HOMO–3	→	LUMO (7.96%)
			HOMO–2	→	LUMO (89.40%)
4	331.90	0.1699	HOMO–3	→	LUMO (87.89%)
			HOMO–2	→	LUMO (3.42%)
5	316.29	0.0304	HOMO–5	→	LUMO (12.58%)
			HOMO–4	→	LUMO (79.33%)

Table S4. Molecular orbital energies (in eV) and molecular orbital distributions (in %) of **15CH** at the ground state (S_0) and first singlet excited state (S_1) optimized geometries in THF

	E (eV)	Car1–H	Car1	naphthyl	Car2	Car2–H
			S_0			
LUMO+3	–0.49	0.0	47.7	4.2	48.0	0.0
LUMO+2	–1.03	0.0	25.7	48.6	25.7	0.0
LUMO+1	–1.26	0.1	2.9	94.0	2.9	0.1
LUMO	–2.31	0.0	6.6	86.8	6.6	0.0
HOMO	–6.78	0.1	4.1	91.7	4.1	0.1
HOMO−1	–7.54	0.0	0.6	98.7	0.6	0.0
HOMO−2	–8.26	0.0	19.1	61.8	19.1	0.0
HOMO−3	–8.54	0.0	47	6.1	46.9	0.0
			S_1			
LUMO+3	–0.61	0.0	5.2	42.4	52.4	0.0
LUMO+2	–1.22	0.0	2.4	92.6	4.7	0.3
LUMO+1	–2.20	0.1	10.9	80.3	8.7	0.0
LUMO	–3.72	0.4	78.7	20.3	0.5	0.0
HOMO	–6.61	0.0	9.3	86.4	4.3	0.1
HOMO−1	–7.59	0.0	2.0	97.3	0.7	0.0
HOMO−2	–8.04	0.0	96.4	3.4	0.2	0.0
HOMO−3	–8.15	0.0	23.9	62.1	13.9	0.0

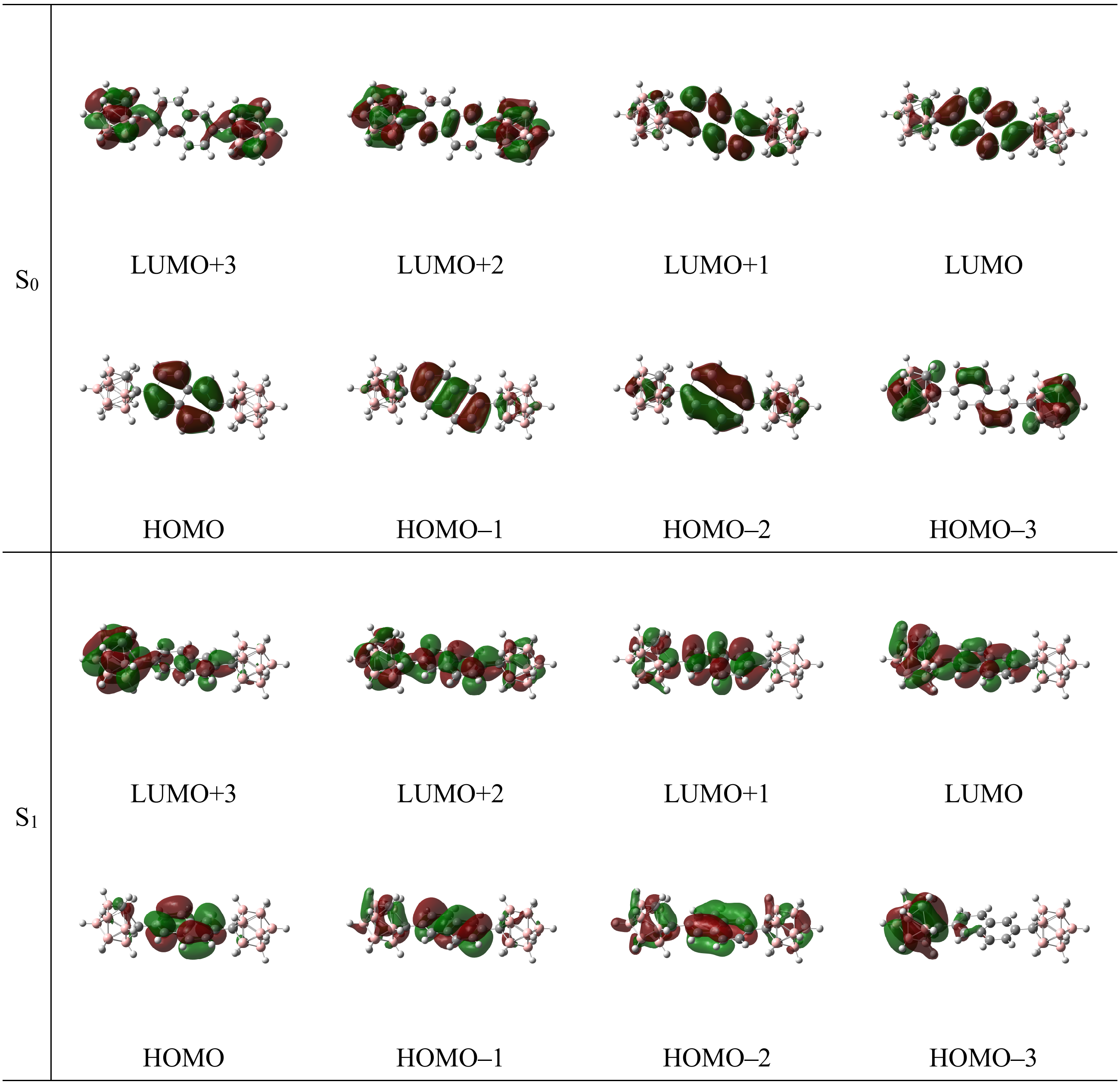

Figure S14. The selected frontier orbitals of **26CH** from TD-B3LYP calculations (Isovalue = 0.04 a.u.) at the ground state (S_0) and first singlet excited state (S_1) optimized geometries in THF.

Table S5. Computed absorption wavelengths (λ_{calc} in nm) and oscillator strengths ($f_{calc.}$) for **26CH** from TD-B3LYP calculations using the B3LYP geometries at the ground state (S_0) and first singlet excited state (S_1) optimized geometries in THF

state	λ_{calc} /nm	f_{calc}	Major contribution
		S_0	
1	301.26	0.0907	HOMO → LUMO (79.72%)
			HOMO → LUMO+1 (8.07%)
2	287.81	0.0201	HOMO–1 → LUMO (39.48%)
			HOMO → LUMO (15.41%)
			HOMO → LUMO+1 (43.79%)
3	243.09	2.3710	HOMO–1 → LUMO (49.49%)
			HOMO → LUMO+1 (44.65%)
4	229.71	0.0000	HOMO–2 → LUMO (9.70%)
			HOMO → LUMO+2 (86.60%)
5	221.53	0.1251	HOMO–1 → LUMO+1 (81.95%)
			HOMO → LUMO+3 (10.11%)
		S_1	
1	550.11	0.3533	HOMO → LUMO (98.41%)
2	340.63	0.0642	HOMO–1 → LUMO (89.44%)
3	312.71	0.1023	HOMO → LUMO+1 (90.42%)
4	286.22	0.0009	HOMO–3 → LUMO (90.43%)
5	278.08	0.1974	HOMO–2 → LUMO (11.74%)
			HOMO–1 → LUMO+1 (23.35%)
			HOMO → LUMO+2 (48.11%)
			HOMO → LUMO+3 (11.94%)

Table S6. Molecular orbital energies (in eV) and molecular orbital distributions (in %) of **26CH** at the ground state (S_0) and first singlet excited state (S_1) optimized geometries in THF

	E (eV)	Car1–H	Car1	naphthyl	Car2	Car2–H
			S_0			
LUMO+3	–0.19	0.1	46.5	6.8	46.6	0.1
LUMO+2	–0.61	0.0	35.6	28.7	35.6	0.0
LUMO+1	–1.60	0.2	8.4	82.8	8.4	0.2
LUMO	–2.19	0.1	6.0	87.8	6.0	0.1
HOMO	–6.75	0.0	3.0	94.0	3.0	0.0
HOMO−1	–7.44	0.1	5.5	88.9	5.5	0.1
HOMO−2	–8.52	0.0	21.8	56.4	21.8	0.0
HOMO−3	–8.87	0.0	44.2	11.6	44.2	0.0
			S_1			
LUMO+3	–1.05	0.1	97.5	2.4	0.0	0.0
LUMO+2	–1.28	0.0	10.5	72.8	16.7	0.0
LUMO+1	–2.17	0.1	23.7	73.2	3.0	0.0
LUMO	–3.63	0.3	76.7	20.7	2.2	0.0
HOMO	–6.60	0.0	5.8	81.8	2.3	0.0
HOMO−1	–7.59	0.1	18.6	77.3	4.0	0.0
HOMO−2	–8.38	0.2	53.2	33.6	13.0	0.0
HOMO−3	–8.43	0.1	80.8	12.7	6.5	0.0

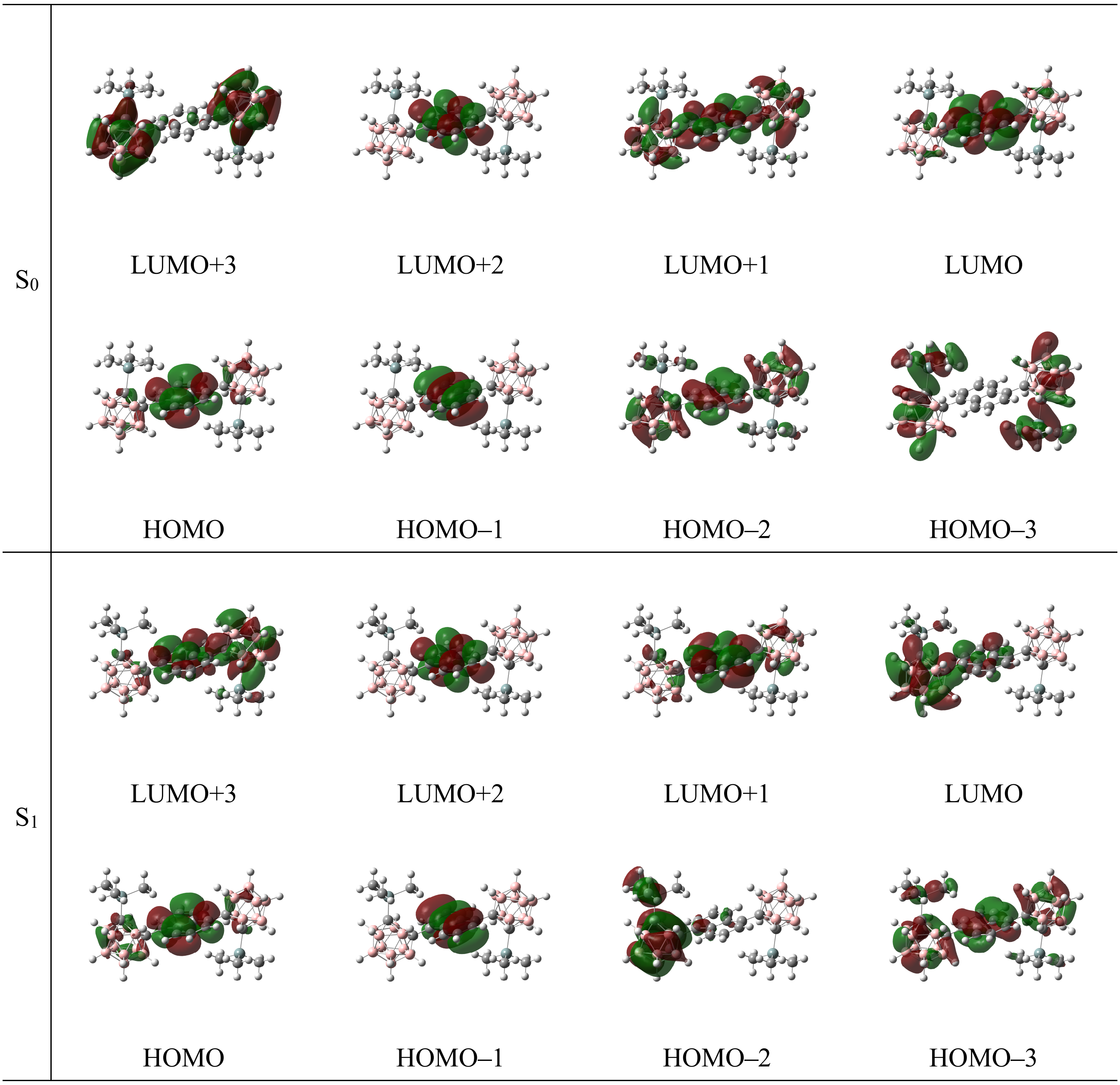

Figure S15. The selected frontier orbitals of **15CS** from TD-B3LYP calculations (Isovalue = 0.04 a.u.) at the ground state (S_0) and first singlet excited state (S_1) optimized geometries in THF.

Table S7. Computed absorption wavelengths (λ_{calc} in nm) and oscillator strengths ($f_{calc.}$) for **15CS** from TD-B3LYP calculations using the B3LYP geometries at the ground state (S_0) and first singlet excited state (S_1) optimized geometries in THF

state	λ_{calc} /nm	f_{calc}	Major contribution
		S_0	
1	314.32	0.4170	HOMO → LUMO (96.52%)
2	288.98	0.0119	HOMO–1 → LUMO (63.00%)
			HOMO → LUMO+2 (35.55%)
3	257.95	0.0000	HOMO → LUMO+1 (92.61%)
4	233.70	0.0000	HOMO–2 → LUMO (89.35%)
5	232.75	0.7593	HOMO–1 → LUMO (32.86%)
			HOMO → LUMO+2 (57.84%)
		S_1	
1	549.59	0.4096	HOMO → LUMO (99.64%)
2	398.35	0.0191	HOMO–1 → LUMO (95.01%)
3	384.06	0.0003	HOMO–2 → LUMO (89.44%)
4	347.37	0.0066	HOMO–4 → LUMO (12.81%)
			HOMO–3 → LUMO (85.23%)
5	334.89	0.1589	HOMO–4 → LUMO (78.81%)
			HOMO–3 → LUMO (13.20%)

Table S8. Molecular orbital energies (in eV) and molecular orbital distributions (in %) of **15CS** at the ground state (S_0) and first singlet excited state (S_1) optimized geometries in THF

	E (eV)	Car1–TMS	Car1	naphthyl	Car2	Car2–TMS
			S_0			
LUMO+3	−0.36	1.7	46.6	3.4	46.6	1.7
LUMO+2	−1.11	1.4	27.8	41.6	7.8	1.4
LUMO+1	−1.19	0.7	2.7	93.3	2.7	0.7
LUMO	−2.27	0.6	7.4	84.0	7.4	0.6
HOMO	−6.67	0.3	4.7	90.0	4.7	0.3
HOMO−1	−7.46	0.5	0.8	97.3	0.8	0.5
HOMO−2	−8.09	1.5	20.7	55.6	20.7	1.5
HOMO−3	−8.30	7.5	42.3	0.4	42.3	7.5
			S_1			
LUMO+3	−0.66	0.5	4.6	42.2	49.5	49.5
LUMO+2	−1.08	0.3	2.7	93.7	2.7	2.7
LUMO+1	−2.13	0.5	8.3	80.6	10.0	10.0
LUMO	−3.64	2.7	80.1	16.7	0.4	0.4
HOMO	−6.50	0.3	8.7	86.2	4.6	4.6
HOMO−1	−7.50	0.3	1.8	96.6	0.9	0.9
HOMO−2	−7.86	8.8	88.1	2.9	0.1	0.1
HOMO−3	−8.00	3.8	23.3	53.8	18.1	18.1

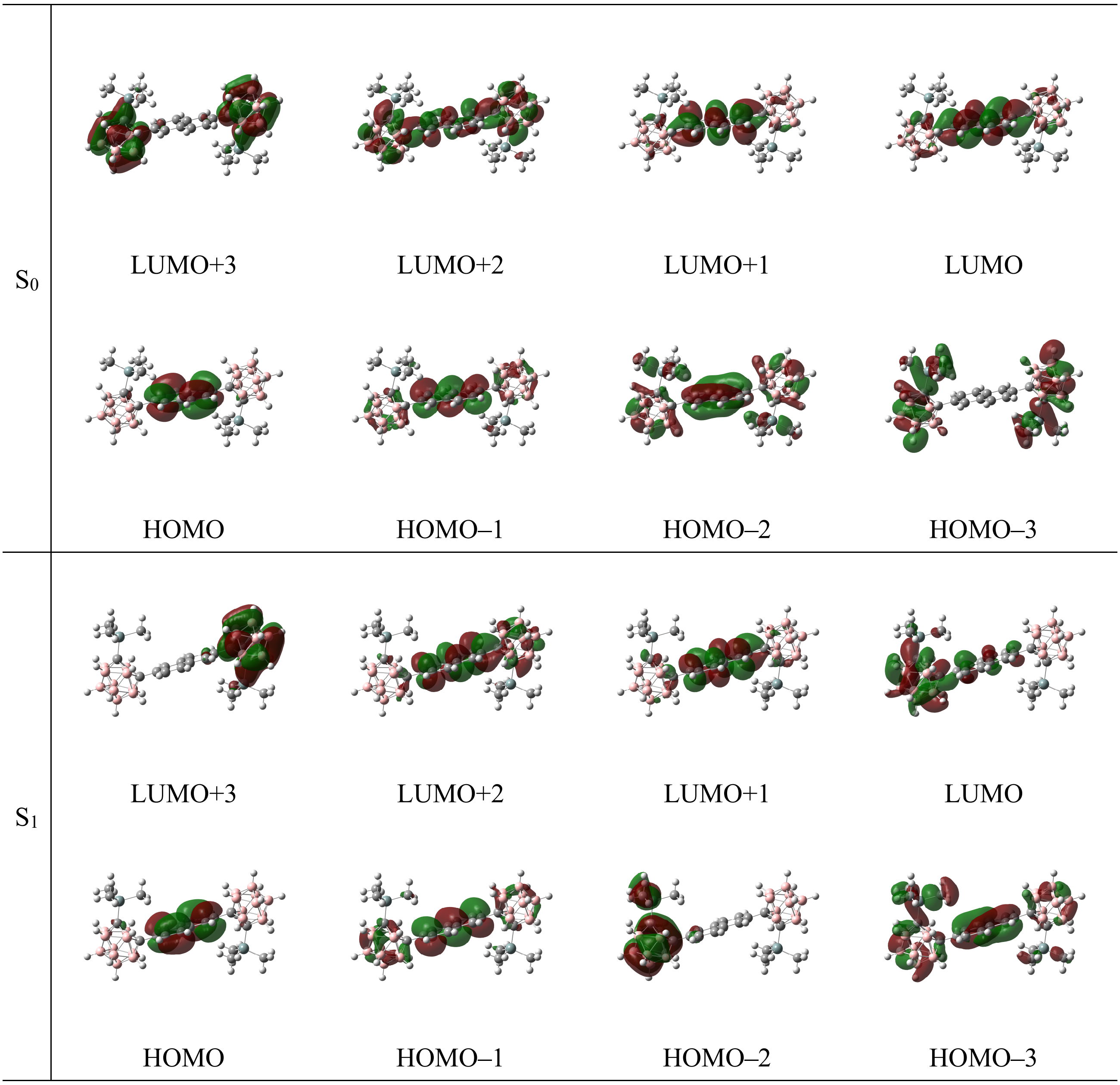

Figure S16. The selected frontier orbitals of **26CS** from TD-B3LYP calculations (Isovalue = 0.04 a.u.) at the ground state (S_0) and first singlet excited state (S_1) optimized geometries in THF.

Table S9. Computed absorption wavelengths (λ_{calc} in nm) and oscillator strengths ($f_{calc.}$) for **26CS** from TD-B3LYP calculations using the B3LYP geometries at the ground state (S_0) and first singlet excited state (S_1) optimized geometries in THF

state	λ_{calc} /nm	f_{calc}	Major contribution
		S_0	
1	317.34	0.1732	HOMO–1 → LUMO (6.15%)
			HOMO → LUMO (83.15%)
2	287.39	0.0614	HOMO–1 → LUMO (48.07%)
			HOMO → LUMO (12.20%)
			HOMO → LUMO+1 (38.67%)
3	244.77	2.3620	HOMO–1 → LUMO (43.18%)
			HOMO → LUMO+1 (51.29%)
4	230.69	0.0000	HOMO–2 → LUMO (14.59%)
			HOMO → LUMO+2 (78.51%)
5	221.08	0.1594	HOMO–1 → LUMO+1 (82.97%)
			HOMO → LUMO+5 (6.79%)
		S_1	
1	560.42	0.4660	HOMO → LUMO (99.39%)
2	404.78	0.1321	HOMO–1 → LUMO (98.10%)
3	382.88	0.0005	HOMO–2 → LUMO (95.44%)
4	343.85	0.0263	HOMO–4 → LUMO (19.44%)
			HOMO–3 → LUMO (78.85%)
5	324.31	0.0006	HOMO–4 → LUMO (87.40%)

Table S10. Molecular orbital energies (in eV) and molecular orbital distributions (in %) of **26CS** at the ground state (S_0) and first singlet excited state (S_1) optimized geometries in THF

	E (eV)	Car1–TMS	Car1	naphthyl	Car2	Car2–TMS
			S_0			
LUMO+3	−0.27	1.9	47.0	2.1	47.0	1.9
LUMO+2	−0.56	2.0	27.4	41.2	27.4	2.0
LUMO+1	−1.49	0.6	5.9	87.0	5.9	0.6
LUMO	−2.21	0.5	7.3	84.3	7.3	0.5
HOMO	−6.74	0.5	2.6	94.0	2.6	0.5
HOMO−1	−7.38	0.6	6.6	85.6	6.6	0.6
HOMO−2	−8.26	6.3	42.0	3.5	42.0	6.3
HOMO−3	−8.28	7.2	42.6	0.4	42.6	7.2
			S_1			
LUMO+3	−0.27	0.0	0.1	3.1	92.9	3.9
LUMO+2	−1.27	0.4	4.5	77.3	16.7	1.2
LUMO+1	−2.09	0.5	6.9	87.3	4.8	0.5
LUMO	−3.63	3.0	81.4	15.0	0.7	0.1
HOMO	−6.53	0.2	3.7	93.5	2.2	0.4
HOMO−1	−7.40	0.7	9.8	82.6	6.5	0.5
HOMO−2	−7.87	8.8	89.3	1.9	0.0	0.0
HOMO−3	−8.16	12.3	84.9	1.9	0.8	0.1

Table S11. Computed emission wavelengths (λ_{calc}), oscillator strengths (f_{calc}), and molecular orbitals of HOMO and LUMO for **15CH** and **26CH** as a function of the dihedral angle (Ψ) between C–C bond axis of the *o*-carborane and the naphthyl plane in each first excited (S_1) state.

15CH

Ψ /°	λ_{calc} /nm	f_{calc}	LUMO	occupation /%		HOMO	occupation /%	
				CB	Nap		CB	Nap
0	695.96	0.0005	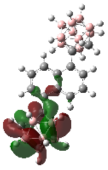	95.91	4.09	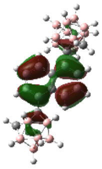	11.07	88.93
15	675.77	0.0383	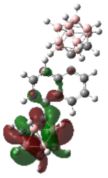	93.95	6.05	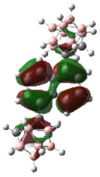	11.31	88.69
30	632.35	0.1277	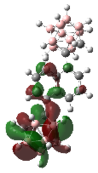	89.92	10.08	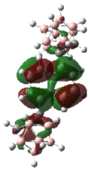	12.22	87.78
45	605.59	0.2473	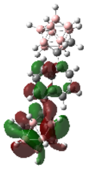	85.35	14.65	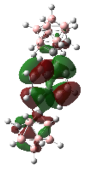	12.87	87.13
60	577.95	0.3522	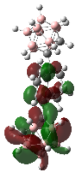	81.53	18.47	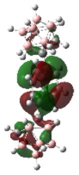	13.13	86.87
75	549.76	0.416	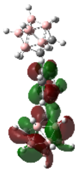	79.3	20.7	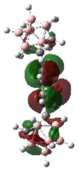	13.2	86.8
90	543.58	0.4329	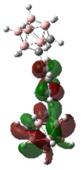	79.14	20.86	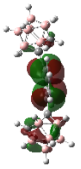	13.39	86.61
105	548.73	0.402	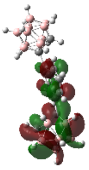	80.8	19.2	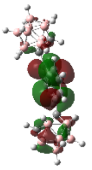	14.09	85.91

120	575.38	0.3615	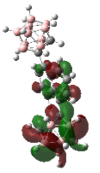	83.94	16.06	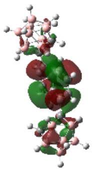	14.13	85.87
135	606.63	0.2203	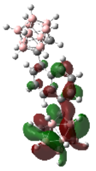	87.74	12.26	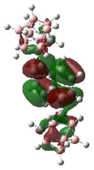	13.17	86.83
150	632.52	0.1151	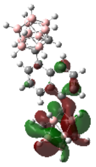	91.76	8.24	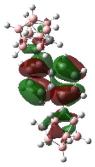	12.25	87.75
165	649.34	0.0244	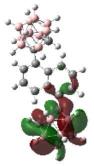	95.14	4.86	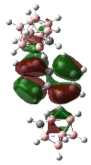	11.59	88.41
180	656.41	0.0005	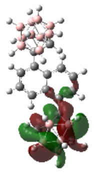	96.07	3.93	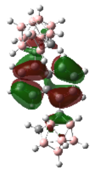	11.5	88.5

26CH

Ψ /°	λ_{calc} /nm	f_{calc}	LUMO	occupation /%		HOMO	occupation /%	
				CB	Nap		CB	Nap
0	696.05	0.0001	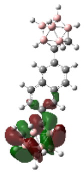	95.84	4.16	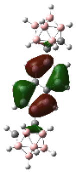	17.03	82.97
15	655.61	0.0471	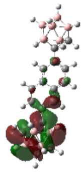	95.65	4.35	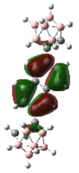	17.11	82.89
30	624.56	0.152	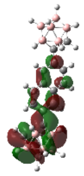	93.05	6.95	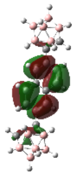	17.35	82.65
45	604.18	0.2383	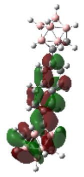	88.24	11.76	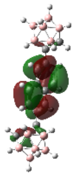	17.69	82.31
60	584.14	0.2794	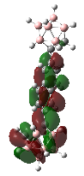	85.21	14.79	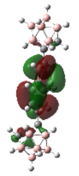	18.02	81.98
75	563.63	0.3167	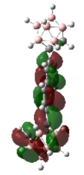	78.17	21.83	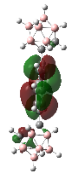	18.18	81.82
90	553.20	0.3458	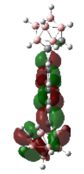	76.97	23.03	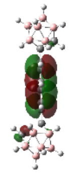	18.07	81.93
105	563.84	0.3173	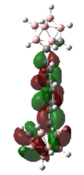	79.65	20.35	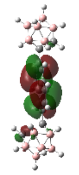	17.78	82.22

120	585.70	0.2695	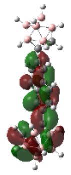	85.98	14.02	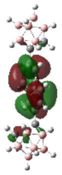	17.55	82.45
135	607.98	0.2031	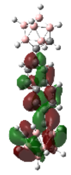	88.82	11.18	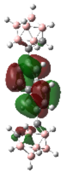	17.5	82.5
150	629.76	0.1424	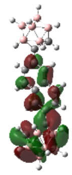	93.79	6.21	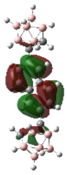	17.53	82.47
165	650.48	0.0367	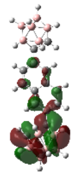	95.54	4.46	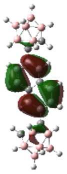	17.53	82.47
180	690.86	0.0001	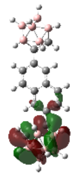	95.8	4.2	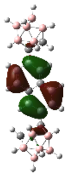	17.51	82.49

Table S12. Cartesian coordinates of the ground state (S_0) fully optimized geometry of **15CH** in THF from B3LYP calculations (in Å)

Atom	X	Y	Z
C	4.228646	0.466736	-1.289619
C	3.277865	0.264475	0.097232
B	4.473437	1.563459	-0.013068
H	4.144199	2.681462	-0.150919
B	5.831165	0.930153	-0.953317
H	6.356615	1.675849	-1.705958
B	5.436432	-0.722285	-1.445042
H	5.696116	-1.111781	-2.531042
B	3.825159	-1.111185	-0.815772
H	3.046588	-1.693978	-1.476153
B	3.905029	-1.069267	0.972858
H	3.163730	-1.708788	1.629948
B	5.259927	-1.714269	0.020337
H	5.502245	-2.875196	0.016029
B	6.515136	-0.445136	-0.062327
H	7.671794	-0.700518	-0.131588
B	5.906950	0.986631	0.821061
H	6.607374	1.760789	1.383904
B	4.288008	0.594530	1.458563
H	3.770907	1.076049	2.406550
B	5.564736	-0.650863	1.426193
H	6.025508	-1.066667	2.437872
C	1.799023	0.672226	0.057298
C	1.526674	2.028480	0.063143
H	2.329439	2.746093	0.117424
C	0.217312	2.523531	-0.014987
H	0.054920	3.595849	-0.034720
C	-0.850056	1.667319	-0.075907
H	-1.829481	2.097791	-0.162835
C	-0.680819	0.258269	-0.031530
C	-4.228409	-0.466600	1.289665
C	-3.277837	-0.264238	-0.097314
B	-4.472949	-1.563607	0.013310
H	-4.143285	-2.681474	0.151300
B	-5.830800	-0.930621	0.953601
H	-6.355915	-1.676378	1.706416
B	-5.436582	0.722033	1.445024
H	-5.696287	1.111613	2.530988
B	-3.825506	1.111381	0.815526
H	-3.047070	1.694542	1.475740
B	-3.905553	1.069149	-0.973100
H	-3.164565	1.708813	-1.630389
B	-5.260568	1.713843	-0.020532
H	-5.503282	2.874688	-0.016385
B	-6.515336	0.444293	0.062467
H	-7.672074	0.699290	0.131809
B	-5.906751	-0.987407	-0.820758
H	-6.606968	-1.761895	-1.383404
B	-4.288013	-0.594851	-1.458493
H	-3.770849	-1.076343	-2.406458
B	-5.565166	0.650107	-1.426185
H	-6.026188	1.065592	-2.437882
C	-1.798894	-0.671633	-0.057438
C	-1.526474	-2.027874	-0.063164
H	-2.329228	-2.745517	-0.117355
C	-0.217099	-2.522885	0.015003
H	-0.054673	-3.595196	0.034831
C	0.850233	-1.666631	0.075856
H	1.829698	-2.097034	0.162841
C	0.680921	-0.257601	0.031373
H	-3.694257	-0.848113	2.150425
H	3.694712	0.848567	-2.150374

Table S13. Cartesian coordinates of the first-excited state (S_1) fully optimized geometry of **15CH** in THF from B3LYP calculations (in Å)

Atom	X	Y	Z
C	4.475795	0.730874	-1.017625
C	3.261941	0.230347	0.001506
B	4.332877	1.557181	0.476531
H	3.923847	2.657261	0.500988
B	5.911493	1.158339	-0.211732
H	6.547389	2.047924	-0.660273
B	5.767833	-0.374853	-1.096292
H	6.309826	-0.518295	-2.136698
B	4.088984	-0.942047	-0.973186
H	3.503235	-1.391836	-1.890578
B	3.742765	-1.245732	0.755108
H	2.919736	-2.019225	1.084437
B	5.328030	-1.649546	0.065120
H	5.652655	-2.777503	-0.098497
B	6.464259	-0.353993	0.539392
H	7.618898	-0.560552	0.712616
B	5.559908	0.850787	1.504482
H	6.041916	1.509751	2.363618
B	3.865036	0.294522	1.629481
H	3.092065	0.559783	2.483018
B	5.203746	-0.884245	1.677282
H	5.433787	-1.484586	2.673896
C	1.830345	0.615257	-0.358686
C	1.566577	1.977704	-0.585873
H	2.377437	2.689166	-0.612091
C	0.276039	2.455047	-0.748186
H	0.107311	3.510508	-0.926152
C	-0.810712	1.583519	-0.638886
H	-1.804416	1.985212	-0.743626
C	-0.625047	0.195094	-0.467080
C	-4.315739	0.002476	1.845726
C	-3.179526	-0.260775	-0.238947
B	-4.235063	-1.274268	0.728988
H	-3.806782	-2.316246	1.100859
B	-5.774742	-0.615749	1.535996
H	-6.326448	-1.214664	2.400675
B	-5.398043	1.148687	1.500855
H	-5.657321	1.950300	2.338227
B	-3.720947	1.093344	0.689305
H	-2.925131	1.895259	1.049483
B	-4.016576	0.806326	-1.142391
H	-3.441611	1.373713	-2.011833
B	-5.263488	1.629554	-0.194842
H	-5.529523	2.752346	-0.484828
B	-6.518532	0.440263	0.295147
H	-7.681393	0.690853	0.343789
B	-5.864480	-1.167023	-0.144823
H	-6.559366	-2.096692	-0.405230
B	-4.387197	-0.959561	-1.086061
H	-4.072708	-1.770004	-1.895501
B	-5.702266	0.241219	-1.245982
H	-6.291613	0.347745	-2.274925
C	-1.754564	-0.699451	-0.418047
C	-1.503966	-2.077722	-0.532708
H	-2.336493	-2.767895	-0.556709
C	-0.207568	-2.571778	-0.605746
H	-0.045291	-3.639378	-0.708020
C	0.895638	-1.720927	-0.539770
H	1.874280	-2.156661	-0.630764
C	0.727732	-0.325041	-0.432866
H	-3.807661	-0.091596	2.796419
H	4.143718	1.261538	-1.901062

Table S14. Cartesian coordinates of the ground state (S_0) fully optimized geometry of **26CH** in THF from B3LYP calculations (in Å)

Atom	X	Y	Z
C	-4.032188	-0.028709	0.051683
C	-4.987015	-1.146751	-0.686506
H	-4.463127	-1.967897	-1.156824
B	-4.805227	0.369663	-1.461164
H	-4.109716	0.425703	-2.412443
B	-4.759729	1.517326	-0.107724
H	-4.065192	2.468491	-0.198812
B	-4.853792	0.609254	1.421383
H	-4.188080	0.930297	2.344317
B	-4.966928	-1.117764	1.030794
H	-4.381173	-1.969734	1.597985
B	-6.463894	-1.388806	0.115525
H	-6.917458	-2.481120	0.121204
B	-6.424486	-0.232100	1.467510
H	-6.964195	-0.465191	2.496186
B	-6.300601	1.406682	0.765116
H	-6.756108	2.359894	1.302433
B	-6.263983	1.255518	-1.018353
H	-6.687889	2.087010	-1.748215
B	-6.369837	-0.473645	-1.409794
H	-6.758640	-0.962661	-2.414034
B	-7.301312	0.170899	-0.040893
H	-8.484202	0.225799	-0.083655
C	-1.677962	0.837369	0.031957
H	-2.070672	1.846934	0.050182
C	-2.536572	-0.244243	0.025376
C	-1.987897	-1.558079	0.007574
H	-2.642612	-2.421669	0.031443
C	-0.628621	-1.757775	-0.016748
H	-0.229764	-2.767897	-0.029849
C	0.271254	-0.660327	-0.014812
C	4.032188	0.028709	-0.051681
C	4.987016	1.146756	0.686499
H	4.463130	1.967904	1.156813
B	4.805230	-0.369654	1.461166
H	4.109720	-0.425687	2.412448
B	4.759729	-1.517325	0.107734
H	4.065191	-2.468490	0.198830
B	4.853789	-0.609263	-1.421379
H	4.188076	-0.930312	-2.344310
B	4.966926	1.117757	-1.030801
H	4.381170	1.969724	-1.597996
B	6.463894	1.388805	-0.115537
H	6.917458	2.481119	-0.121223
B	6.424483	0.232090	-1.467514
H	6.964191	0.465175	-2.496192
B	6.300600	-1.406687	-0.765109
H	6.756105	-2.359903	-1.302421
B	6.263985	-1.255512	1.018359
H	6.687892	-2.086999	1.748225
B	6.369840	0.473654	1.409789
H	6.758645	0.962676	2.414025
B	7.301312	-0.170899	0.040891
H	8.484202	-0.225800	0.083650
C	1.677963	-0.837369	-0.031955
H	2.070672	-1.846933	-0.050180
C	2.536573	0.244243	-0.025374
C	1.987898	1.558080	-0.007573
H	2.642612	2.421670	-0.031443
C	0.628621	1.757775	0.016749
H	0.229764	2.767897	0.029850
C	-0.271254	0.660327	0.014814

Table S15. Cartesian coordinates of the first-excited state (S_1) fully optimized geometry of **26CH** in THF from B3LYP calculations (in Å)

Atom	X	Y	Z
C	-4.051786	-0.056398	0.072026
C	-5.022886	-1.103347	-0.766182
H	-4.503838	-1.899237	-1.283805
B	-4.810421	0.459336	-1.427141
H	-4.101559	0.578968	-2.362359
B	-4.761781	1.506184	0.007675
H	-4.061935	2.457345	0.001335
B	-4.903583	0.498433	1.471516
H	-4.255972	0.747192	2.429677
B	-5.029684	-1.192359	0.954440
H	-4.475119	-2.092532	1.475470
B	-6.509502	-1.378757	-0.003726
H	-6.985067	-2.459200	-0.078859
B	-6.484605	-0.318692	1.426422
H	-7.048215	-0.615914	2.427099
B	-6.321080	1.361317	0.847789
H	-6.773901	2.283860	1.442006
B	-6.257503	1.335585	-0.942147
H	-6.658916	2.227135	-1.613899
B	-6.388614	-0.355661	-1.457328
H	-6.775105	-0.763369	-2.498374
B	-7.328256	0.203757	-0.060660
H	-8.511251	0.281181	-0.114755
C	-1.668763	0.836104	0.045498
H	-2.064734	1.843628	0.070379
C	-2.579056	-0.285043	0.045769
C	-2.023062	-1.560880	0.018693
H	-2.655554	-2.441440	0.030967
C	-0.629469	-1.757248	-0.015392
H	-0.241040	-2.770567	-0.036461
C	0.281250	-0.661575	-0.016737
C	4.051786	0.056398	-0.072025
C	5.022887	1.103347	0.766182
H	4.503838	1.899237	1.283805
B	4.810421	-0.459337	1.427140
H	4.101559	-0.578968	2.362359
B	4.761781	-1.506184	-0.007675
H	4.061935	-2.457345	-0.001335
B	4.903583	-0.498433	-1.471516
H	4.255971	-0.747192	-2.429677
B	5.029683	1.192359	-0.954440
H	4.475119	2.092532	-1.475470
B	6.509502	1.378757	0.003726
H	6.985067	2.459200	0.078859
B	6.484605	0.318692	-1.426422
H	7.048215	0.615914	-2.427100
B	6.321080	-1.361317	-0.847789
H	6.773900	-2.283860	-1.442007
B	6.257504	-1.335585	0.942146
H	6.658916	-2.227136	1.613899
B	6.388615	0.355661	1.457328
H	6.775106	0.763369	2.498373
B	7.328256	-0.203757	0.060659
H	8.511251	-0.281181	0.114754
C	1.668763	-0.836104	-0.045498
H	2.064734	-1.843628	-0.070379
C	2.579056	0.285043	-0.045769
C	2.023062	1.560880	-0.018692
H	2.655554	2.441440	-0.030966
C	0.629469	1.757248	0.015393
H	0.241040	2.770567	0.036461
C	-0.281250	0.661575	0.016738

Table S16. Cartesian coordinates of the ground state (S_0) fully optimized geometry of **15CS** in THF from B3LYP calculations (in Å)

Atom	X	Y	Z
C	-4.505311	0.253285	0.082247
C	-3.171109	-0.820796	-0.329576
B	-4.464741	-0.491949	-1.462207
H	-4.290810	0.180141	-2.410468
B	-5.971532	-0.372427	-0.550712
H	-6.771430	0.415750	-0.927163
B	-5.576842	-0.556013	1.154857
H	-6.101357	0.089844	1.994385
B	-3.825390	-0.766749	1.270651
H	-3.214201	-0.262924	2.140255
B	-3.406591	-2.315002	0.464418
H	-2.444921	-2.917145	0.791236
B	-4.899949	-2.174742	1.405732
H	-5.015539	-2.732660	2.445430
B	-6.247149	-1.932354	0.257085
H	-7.347838	-2.315531	0.474129
B	-5.554430	-1.878396	-1.389493
H	-6.138056	-2.218637	-2.363615
B	-3.800595	-2.126495	-1.254463
H	-3.090109	-2.565603	-2.091887
B	-4.907296	-3.010647	-0.175139
H	-5.020727	-4.187349	-0.268427
Si	-4.283219	2.190363	0.219684
C	-2.738292	2.629395	1.204789
H	-2.705252	3.718229	1.328525
H	-1.815030	2.325225	0.706243
H	-2.747722	2.185350	2.204296
C	-4.200928	2.937347	-1.508924
H	-4.183109	4.029830	-1.421718
H	-5.072608	2.667257	-2.112369
H	-3.301633	2.634809	-2.051777
C	-5.804355	2.852524	1.114877
H	-5.763256	3.947984	1.116336
H	-5.848056	2.518959	2.155125
H	-6.735174	2.553288	0.625125
C	-1.782619	-0.267328	-0.667118
C	-1.613444	0.242822	-1.942545
H	-2.431512	0.215981	-2.645498
C	-0.405373	0.820119	-2.359774
H	-0.333702	1.243560	-3.356453
C	0.674863	0.864796	-1.518332
H	1.566051	1.351767	-1.866268
C	0.631609	0.288589	-0.221208
C	4.505307	-0.253287	-0.082247
C	3.171112	0.820805	0.329563
B	4.464745	0.491969	1.462197
H	4.290815	-0.180107	2.410468
B	5.971534	0.372427	0.550698
H	6.771429	-0.415752	0.927154
B	5.576843	0.555993	-1.154872
H	6.101355	-0.089876	-1.994390
B	3.825391	0.766732	-1.270665
H	3.214197	0.262896	-2.140260
B	3.406600	2.314999	-0.464451
H	2.444933	2.917144	-0.791274
B	4.899955	2.174720	-1.405767
H	5.015544	2.732625	-2.445473
B	6.247156	1.932342	-0.257119
H	7.347846	2.315511	-0.474170
B	5.554441	1.878409	1.389461
H	6.138071	2.218661	2.363577
B	3.800606	2.126515	1.254432
H	3.090123	2.565637	2.091851
B	4.907308	3.010646	0.175094
H	5.020745	4.187349	0.268364
Si	4.283206	-2.190366	-0.219645
C	2.738121	-2.629436	-1.204486
H	2.705166	-3.718260	-1.328336
H	1.814934	-2.325414	-0.705714
H	2.747300	-2.185278	-2.203945
C	4.201206	-2.937327	1.508987
H	4.183404	-4.029813	1.421808
H	5.072971	-2.667198	2.112292
H	3.301989	-2.634803	2.051976
C	5.804204	-2.852516	-1.115080
H	5.763119	-3.947976	-1.116519
H	5.847732	-2.518963	-2.155339
H	6.735098	-2.553262	-0.625483
C	1.782622	0.267342	0.667109
C	1.613451	-0.242818	1.942533
H	2.431520	-0.215980	2.645485
C	0.405381	-0.820119	2.359759
H	0.333711	-1.243568	3.356435
C	-0.674858	-0.864789	1.518319
H	-1.566047	-1.351758	1.866254
C	-0.631605	-0.288573	0.221198

Table S17. Cartesian coordinates of the first-excited state (S_1) fully optimized geometry of **15CS** in THF from B3LYP calculations (in Å)

Atom	X	Y	Z
C	-4.510063	0.252118	0.082637
C	-3.169819	-0.821149	-0.328989
B	-4.463570	-0.493194	-1.461367
H	-4.293198	0.175690	-2.411389
B	-5.972011	-0.379122	-0.551588
H	-6.777798	0.402122	-0.928890
B	-5.577886	-0.560892	1.153559
H	-6.108220	0.079469	1.993509
B	-3.825664	-0.765760	1.270417
H	-3.218687	-0.263565	2.142618
B	-3.402352	-2.314709	0.465751
H	-2.441973	-2.917256	0.794659
B	-4.896534	-2.177522	1.404847
H	-5.013908	-2.738138	2.444107
B	-6.242840	-1.939253	0.256053
H	-7.343486	-2.328352	0.472189
B	-5.549449	-1.883846	-1.388851
H	-6.133518	-2.228857	-2.362576
B	-3.796177	-2.127287	-1.252925
H	-3.087646	-2.569053	-2.090652
B	-4.900673	-3.013463	-0.174663
H	-5.013423	-4.191926	-0.267717
Si	-4.289316	2.197597	0.219080
C	-2.747224	2.627514	1.209394
H	-2.716115	3.716586	1.330292
H	-1.823585	2.321785	0.712557
H	-2.763789	2.184206	2.209119
C	-4.206695	2.933516	-1.512035
H	-4.182214	4.025604	-1.422880
H	-5.082861	2.668406	-2.111224
H	-3.310807	2.624683	-2.056878
C	-5.815433	2.847424	1.112487
H	-5.771523	3.942602	1.116674
H	-5.858789	2.510224	2.151625
H	-6.744238	2.551373	0.616765
C	-1.782223	-0.266499	-0.666805
C	-1.614986	0.242424	-1.943181
H	-2.431650	0.219150	-2.647674
C	-0.406273	0.816452	-2.362951
H	-0.335475	1.238074	-3.359927
C	0.674493	0.859017	-1.521757
H	1.565626	1.341565	-1.875783
C	0.631648	0.286914	-0.222791
C	4.510115	-0.251946	-0.082543
C	3.169586	0.820965	0.328848
B	4.463327	0.493527	1.461390
H	4.293108	-0.175306	2.411474
B	5.971864	0.379754	0.551756
H	6.777807	-0.401253	0.929220
B	5.577839	0.561217	-1.153467
H	6.108414	-0.079104	-1.993293
B	3.825582	0.765607	-1.270521
H	3.218795	0.263126	-2.142693
B	3.401789	2.314511	-0.466054
H	2.441277	2.916758	-0.795107
B	4.896087	2.177644	-1.405000
H	5.013392	2.738180	-2.444311
B	6.242349	1.939861	-0.256062
H	7.342910	2.329226	-0.472149
B	5.548828	1.884467	1.388789
H	6.132713	2.229748	2.362529
B	3.795495	2.127415	1.252682
H	3.086768	2.569099	2.090286
B	4.899854	3.013758	0.174413
H	5.012266	4.192264	0.267338
Si	4.289911	-2.197497	-0.218769
C	2.747827	-2.628058	-1.208807
H	2.717184	-3.717147	-1.329686
H	1.824152	-2.322745	-0.711784
H	2.764001	-2.184763	-2.208544
C	4.207730	-2.933153	1.512481
H	4.183618	-4.025268	1.423546
H	5.083872	-2.667609	2.111519
H	3.311790	-2.624528	2.057364
C	5.816125	-2.846979	-1.112251
H	5.772538	-3.942171	-1.116285
H	5.859261	-2.509910	-2.151439
H	6.744897	-2.550587	-0.616671
C	1.782064	0.266073	0.666558
C	1.614864	-0.243010	1.942875
H	2.431519	-0.219728	2.647378
C	0.406210	-0.817213	2.362570
H	0.335462	-1.239006	3.359477
C	-0.674573	-0.859702	1.521391
H	-1.565672	-1.342365	1.875349
C	-0.631777	-0.287420	0.222500

Table S18. Cartesian coordinates of the ground state (S_0) fully optimized geometry of **26CS** in THF from B3LYP calculations (in Å)

Atom	X	Y	Z
Si	4.761969	2.240198	-0.005380
C	5.162880	0.318187	0.030340
C	3.937919	-0.885219	-0.113856
C	3.729240	2.700822	1.499913
H	3.564622	3.784375	1.486101
H	4.239902	2.454239	2.435412
H	2.750132	2.215083	1.503620
C	3.869664	2.685694	-1.602009
H	3.687878	3.766674	-1.601759
H	2.903905	2.184697	-1.704611
H	4.473778	2.451403	-2.483429
C	6.416874	3.137669	0.063529
H	6.222136	4.216070	0.086576
H	7.036625	2.928745	-0.812550
H	6.990513	2.882363	0.958668
C	1.658998	-0.330713	0.809314
H	2.053823	-0.411891	1.814857
C	2.481435	-0.514659	-0.287305
C	1.913001	-0.442274	-1.592389
H	2.537015	-0.613657	-2.460093
C	0.577884	-0.167837	-1.769265
H	0.164453	-0.117914	-2.772080
C	0.278239	-0.043539	0.656172
B	4.701815	-0.615353	1.405306
H	4.104674	-0.089553	2.272887
B	6.401841	-0.263920	1.061161
H	6.955395	0.521444	1.751690
B	6.606212	-0.258908	-0.693899
H	7.304218	0.528479	-1.233750
B	5.029085	-0.603135	-1.418302
H	4.638854	-0.069877	-2.393055
B	4.552047	-2.251813	-0.941916
H	3.797247	-2.841455	-1.636169
B	6.246252	-1.884909	-1.305802
H	6.764791	-2.298488	-2.289843
B	7.104254	-1.679616	0.248193
H	8.253459	-1.947223	0.380439
B	5.915108	-1.894848	1.564449
H	6.196784	-2.314195	2.638298
B	4.348513	-2.259773	0.823218
H	3.454591	-2.852684	1.321576
B	5.833409	-2.910192	0.097466
H	6.051645	-4.077301	0.117728
Si	-4.761970	-2.240198	0.005380
C	-5.162880	-0.318187	-0.030340
C	-3.937918	0.885219	0.113856
C	-3.729241	-2.700823	-1.499913
H	-3.564623	-3.784376	-1.486101
H	-4.239903	-2.454239	-2.435412
H	-2.750133	-2.215084	-1.503619
C	-3.869666	-2.685694	1.602009
H	-3.687880	-3.766674	1.601759
H	-2.903907	-2.184698	1.704612
H	-4.473780	-2.451403	2.483429
C	-6.416875	-3.137668	-0.063530
H	-6.222138	-4.216069	-0.086576
H	-7.036627	-2.928743	0.812550
H	-6.990514	-2.882362	-0.958669
C	-1.658997	0.330711	-0.809315
H	-2.053823	0.411890	-1.814857
C	-2.481435	0.514658	0.287305
C	-1.913001	0.442273	1.592389
H	-2.537015	0.613656	2.460092
C	-0.577884	0.167835	1.769265
H	-0.164453	0.117913	2.772080
C	-0.278238	0.043538	-0.656173
B	-4.701815	0.615353	-1.405306
H	-4.104675	0.089553	-2.272887
B	-6.401841	0.263920	-1.061161
H	-6.955395	-0.521442	-1.751689
B	-6.606212	0.258910	0.693899
H	-7.304218	-0.528477	1.233750
B	-5.029084	0.603135	1.418302
H	-4.638854	0.069877	2.393054
B	-4.552046	2.251813	0.941916
H	-3.797246	2.841454	1.636169
B	-6.246251	1.884910	1.305803
H	-6.764789	2.298489	2.289843
B	-7.104253	1.679617	-0.248192
H	-8.253458	1.947224	-0.380439
B	-5.915108	1.894849	-1.564449
H	-6.196783	2.314196	-2.638298
B	-4.348512	2.259773	-0.823218
H	-3.454590	2.852684	-1.321577
B	-5.833407	2.910193	-0.097465
H	-6.051643	4.077302	-0.117727

Table S19. Cartesian coordinates of the first-excited state (S_1) fully optimized geometry of **26CS** in THF from B3LYP calculations (in Å)

Atom	X	Y	Z
Si	5.433342	2.286678	0.074544
C	5.622348	0.406691	0.067166
C	3.775116	-1.099257	-0.183500
C	3.610606	2.753378	-0.132868
H	3.499974	3.843558	-0.114266
H	2.993737	2.341751	0.672628
H	3.206088	2.394593	-1.084708
C	6.424022	3.023693	-1.357350
H	6.285464	4.110171	-1.394909
H	6.103146	2.611684	-2.319801
H	7.494716	2.824367	-1.248787
C	6.060678	2.976531	1.719261
H	5.913630	4.061946	1.756229
H	7.127803	2.776558	1.857572
H	5.525699	2.537199	2.567714
C	1.491807	-0.591624	0.771212
H	1.880649	-0.771470	1.767336
C	2.344727	-0.723835	-0.339090
C	1.793685	-0.505714	-1.610913
H	2.422457	-0.604113	-2.487414
C	0.444139	-0.152186	-1.784402
H	0.060516	0.012569	-2.785831
C	0.130800	-0.227451	0.629250
B	4.705599	-0.574270	1.158786
H	4.135974	-0.036513	2.054953
B	6.552682	-0.476303	1.068674
H	7.201760	0.101362	1.880347
B	6.766176	-0.453323	-0.710107
H	7.589326	0.145081	-1.325176
B	4.992646	-0.541667	-1.242057
H	4.659603	0.024811	-2.234194
B	4.452401	-2.324968	-1.020132
H	3.762591	-2.892261	-1.804970
B	6.150704	-1.985301	-1.344809
H	6.631173	-2.407143	-2.349563
B	7.052882	-1.958229	0.208262
H	8.160748	-2.378169	0.336613
B	5.807385	-2.026110	1.497049
H	6.030954	-2.478008	2.575883
B	4.238589	-2.340669	0.766592
H	3.377722	-2.909752	1.359261
B	5.691813	-3.054405	0.025065
H	5.850747	-4.235376	0.028675
Si	-5.059908	-2.152998	0.043160
C	-5.340111	-0.206871	-0.024755
C	-4.053663	0.924734	0.114325
C	-3.868110	-2.676671	-1.316925
H	-3.779615	-3.768771	-1.286972
H	-4.234369	-2.401456	-2.310076
H	-2.863292	-2.264148	-1.193439
C	-4.405186	-2.637444	1.740167
H	-4.320173	-3.729732	1.772345
H	-3.415077	-2.225065	1.951762
H	-5.083470	-2.333861	2.542753
C	-6.741495	-2.947183	-0.244736
H	-6.621215	-4.034696	-0.180926
H	-7.476050	-2.647268	0.507393
H	-7.145405	-2.712903	-1.233173
C	-1.771806	0.349102	-0.821482
H	-2.150604	0.527992	-1.820340
C	-2.622569	0.477801	0.290075
C	-2.072840	0.279619	1.565639
H	-2.686345	0.408667	2.447476
C	-0.726370	-0.074108	1.734834
H	-0.341872	-0.223826	2.738118
C	-0.411106	-0.004655	-0.673984
B	-4.816837	0.681798	-1.409303
H	-4.235156	0.108875	-2.258598
B	-6.535775	0.439523	-1.070987
H	-7.132490	-0.317529	-1.755190
B	-6.747136	0.465834	0.684669
H	-7.492065	-0.273383	1.229799
B	-5.158852	0.727237	1.417451
H	-4.804764	0.184052	2.400235
B	-4.580706	2.339457	0.925066
H	-3.787594	2.882214	1.614576
B	-6.295613	2.076726	1.280687
H	-6.794170	2.531175	2.256081
B	-7.153781	1.904601	-0.277545
H	-8.283307	2.239411	-0.419570
B	-5.947307	2.032075	-1.589861
H	-6.195386	2.455552	-2.669509
B	-4.366577	2.310519	-0.841367
H	-3.431200	2.837111	-1.338871
B	-5.812174	3.059352	-0.132440
H	-5.957588	4.236543	-0.169316
